# Unraveling cerebrovascular involvement in EGPA through digital subtraction angiography: case presentation and systematic literature review

**DOI:** 10.1186/s41927-025-00518-7

**Published:** 2025-07-01

**Authors:** Viren Vasandani, Sean O’Leary, Ronak Gandhi, Elena Diller, Giri Movva, John Broussard, Vijaya Murthy

**Affiliations:** 1 John Sealy School of Medicine, University of Texas Medical Branch, 301 University Blvd, Galveston, TX 77550 USA; 2 Department of Rheumatology, University of Texas Medical Branch, Galveston, TX USA; 3 Department of Internal Medicine, University of Texas Medical Branch, Galveston, TX USA; 4 Department of Pathology, University of Texas Medical Branch, Galveston, TX USA

**Keywords:** Eosinophilic granulomatosis with polyangiitis, Churg-strauss syndrome, Central nervous system, Cerebrovascular, Diagnostic imaging, Digital subtraction angiography, Vessel inflammation

## Abstract

**Objectives:**

Eosinophilic granulomatosis with polyangiitis (EGPA) involves systemic inflammation of small to medium vessels, with central nervous system (CNS) involvement being rare. While CT (computed tomography) and MRI (magnetic resonance imaging) are standard for diagnosing CNS involvement, digital subtraction angiography (DSA) is infrequently used. We present a unique EGPA case with CNS involvement and review EGPA CNS vascular variations.

**Methods:**

We present a case of EGPA with CNS involvement, alongside a systematic review of the literature following PRISMA guidelines, querying three databases (PubMed/MEDLINE, SCOPUS, and Science Direct) up to September 2023 for case reports and series on EGPA with CNS involvement.

**Results:**

A 43-year-old presented with wheezing, multifocal neuropathy, leukocytosis, eosinophilia, positive ANA, and elevated CRP. Imaging revealed lung abnormalities. CT and MRI showed cerebral infarcts. CTA was negative, whereas DSA revealed bilateral segmental narrowing of anterior cerebral artery (ACA) branches and middle cerebral artery (MCA) branches. EGPA was confirmed, and treatment with steroids, cyclophosphamide, and azathioprine, led to remission. A systematic literature review of 27 EGPA cases with CNS involvement found a mean age 54.22 years, with common symptoms including extremity weakness (*n* = 8) and paresthesia (*n* = 5). Imaging techniques included MRI (*n* = 21), CT (*n* = 11), angiogram (*n* = 8), MRA (*n* = 4), CTA (*n* = 4), and MRV (*n* = 2), revealing stenosis of the bilateral ACA, vertebral artery, MCA, and basilar artery.

**Conclusion:**

Our findings suggest a potentially novel role for angiographic imaging in the comprehensive assessment of cerebrovascular involvement in EGPA.

## Introduction

Eosinophilic granulomatosis with polyangiitis (EGPA), formerly known as Churg-Strauss syndrome, is a condition characterized by systemic small and medium-vessel inflammation. EGPA presents in three phases: a prodromal phase characterized by the onset of asthma, allergic rhinitis, and sinusitis; an eosinophilic phase characterized by an increase in the peripheral eosinophil count and eosinophilic organ damage without evidence of vasculitis; and a vasculitic phase characterized by vascular or extravascular granulomatosis alongside constitutional symptoms such as fatigue and weight loss [1, 2].

In 1990, the American College of Rheumatology (ACR) created classification guidelines for EGPA. Using these guidelines, EGPA can be confirmed if four of the following six criteria are met: the presence of asthma, an eosinophil count exceeding 10%, neuropathy symptoms, evidence of pulmonary infiltrates, paranasal sinus abnormalities, and biopsy-proven extravascular eosinophils [3]. In 2022, the ACR, alongside the European Alliance of Associations for Rheumatology (EULAR), revised the classification criteria to include the following clinical, laboratory, biopsy components and associated scores: obstructive airway disease (+ 3), nasal polyps (+ 3), mononeuritis multiplex (+ 1), blood eosinophils count ≥ 1 × 109/L (+ 5), extravascular eosinophilic-predominant inflammation on biopsy (+ 2), positive cANCA or anti-PR3 antibody (-3), and hematuria (-1) [4]. A score ≥ 6 on the 2022 guidelines is sufficient for EGPA classification. The severity of EGPA can be gauged using the Five Factor Score (FFS) consisting of the following components: cardiomyopathy, CNS involvement, pronounced GI tract symptoms, renal failure (with a Cr > 1.58 mg/dL), and significant proteinuria (> 1 g/d). The presence of two or more of these factors suggests a severe prognosis [5].

EGPA in the vasculitic phase can affect the peripheral nervous system (PNS), central nervous system (CNS), kidneys, and skin [6, 7]. CNS involvement is a rare complication of EGPA, occurring in less than 5% of cases [8–10]. Primary neurological manifestations include ischemic brain lesions, brain hemorrhages, vision loss, and cranial nerve dysfunction. Typically, computerized tomography (CT) and/or magnetic resonance imaging (MRI), is/are performed to view cerebrovascular dysfunction [10]. Specific patterns of cerebrovascular vasculitis imaged through angiography are poorly characterized in the literature. Here, we present a previously unreported variation of EGPA cerebrovascular pathology, as well as a systematic review of EGPA intracranial vascular variations in the literature. Recognizing such patterns is critical for improving diagnostic accuracy, particularly in patients with inconclusive non-invasive imaging, and may facilitate earlier initiation of appropriate immunosuppressive therapy to prevent irreversible neurological damage.

## Case report description

### History

A 43-year-old Hispanic woman with a past medical history of hypertension, poorly controlled asthma and rhinosinusitis since childhood, and subclinical hypothyroidism presented to an outside hospital with a 3-week history of severe and progressive left upper quadrant abdominal pain. Medication history prior to admission included albuterol, fluticasone, ipratropium, carbamazepine, promethazine, hydrochlorothiazide, and propranolol. Further history revealed that the patient had suffered from recurrent multifocal “pneumonia” refractory to antibiotics, severe fatigue, and unintentional weight loss for the past 10 months. She also reported nausea, vomiting, a recent rash characterized by pinpoint lesions on her palms and soles, and a 3-month history of migratory burning pain and numbness in the extremities. The patient was incarcerated at the time of symptom onset and had no known occupational exposures to toxins or allergens. She denied any fever, chills, cough, hemoptysis, joint pain, malar rash, or family history of cancer and autoimmune conditions.

### Physical exam

Pulmonary examination revealed both inspiratory and expiratory wheezing with no respiratory distress. There was visible bruising on the left upper extremity. Neurologic examination showed cranial nerves II-XII to be intact with numbness and burning pain present in the right 4th and 5th toes, on the plantar aspect of the left foot’s toes, and on the palmar aspect of the 2nd digit on the right hand, progressing from the distal fingertip to the metacarpophalangeal (MCP) joint. Both patellar and Achilles reflexes were slightly diminished. Physical exam did not reveal hepatosplenomegaly, lymphadenopathy, or other systemic findings.

### Chart review

Imaging and laboratory studies from the outside hospital were reviewed prior to the patient’s transfer and admission to the University of Texas Medical Branch (UTMB). CT abdomen and pelvis revealed no acute abdominal or pelvic abnormalities but did reveal periumbilical hernias alongside ground glass lung opacities bilaterally with a right lower lobe nodule. Initial laboratory findings revealed leukocytosis ranging from 18.08 × 10^3/uL to 40.61 × 10^3/uL with an eosinophil count ranging from 9.36 × 10^3/uL to 31.81 × 10^3/uL (51.8 − 78.3%) respectively over the previous 2 months. Thrombocytosis ranged from 403 × 10^3/uL to 583 × 10^3/uL over the same time period. ANA was positive at 1:320 with a speckled pattern, CRP was elevated at 6.7 mg/dL, and antineutrophil cytoplasmic antibodies (ANCA) was found to be negative. TPO antibodies were positive at 158.9 WHO Units (Table 1).

**Table 1:** Lab values for patient prior to admission, and repeat values after admission, significant lab values marked with *

	Prior to admission (min - max)	Admission	Reference range
White blood cell count	5.72 × 10^3^ − 40.61 × 10^3^/µL*	22.13 × 10^3^/µL*	3.8–10.7 × 10^3^/µL
Red blood cell count	4.56 × 10^6^ − 4.83 × 10^6^/µL	3.78 × 10^3^/µL*	4.5–6.2 × 10^6^/µL
Haemoglobin	12.5–13.7 g/dL*	11.0 × 10 g/dL*	13.5–18.0 g/dL
Hematocrit	39.8 − 42.9%	33.1 × 10%*	39–51%
Mean corpuscular volume	87.3–89.9 fL	87.6 fL	80.0–98.0 fL
Absolute neutrophils	-	4.46 × 10^3^/L*	1.88–0.06 × 10^3^/µL
Absolute eosinophils	0.26 × 10^3^ − 31.81 × 10^3^/µL*	14.70 × 10^3^/µL*	0.03–0.39 × 10^3^/µL
Absolute basophils	0.04 × 10^3^ − 0.13 × 10^3^/µL	0.08 × 10^3^/µL	0.01–0.07 × 10^3^/µL
Absolute lymphocytes	2.13 × 10^3^ − 3.97 × 10^3^/µL	2.23 × 10^3^/µL	1.32–3.29 × 10^9^/µL
Absolute monocytes	0.65 × 10^3^ − 0.97 × 10^3^/µL	0.59 × 10^3^/µL	0.33–0.92 × 10^3^/µL
Perinuclear anti-neutrophil cytoplasmic antibodies (p-ANCA)	-	< 0.6 U/mL	< 2 U/mL
Anti-myeloperoxidase (α-MPO)	-	< 1.0 AI	< 1.0 AI
Blood urea nitrogen (BUN)	-	8 mg/dL	7–18 mg/dL
Creatinine	-	0.62 mg/dL	0.70–1.30 mg/dL
C reactive protein	-	7.2 mg/dL*	0.0–0.5 mg/dL
Rheumatoid factor	-	417 IU/mL*	< 14 IU/mL

### Investigations

#### Laboratory

Upon admission, the patient had an elevated white blood cell count of 22.13 × 10³/µL, a reduced hemoglobin level at 11.0 g/dL, and a hematocrit value of 33.1%. Furthermore, there was a significant increase in the absolute neutrophil count at 4.46 × 10³/L and a high absolute eosinophil count of 14.70 × 10³/µL. Additionally, the patient displayed elevated CRP of 7.2 mg/dL and a high rheumatoid factor level of 417 IU/mL (Table 1).

The remaining rheumatologic, infectious, and hematologic tests were unremarkable. Of note, ANCA, MPO, and repeat ANA studies ordered during hospitalization returned negative.

#### Bone marrow

Eosinophilia, in combination with significant unexpected weight loss, led to the suspicion of eosinophilic leukemia as a potential diagnosis. A bone marrow biopsy was ordered for further evaluation. Results showed normocellular bone marrow (~ 70% cellularity) with progressive trilineage hematopoiesis. Increased eosinophils and eosinophilic precursors with hypersegmented neutrophils were also observed. No morphological dysplasia was noted in any of the cell lines, and blasts were not increased. These results were non-diagnostic for eosinophilic leukemia, warranting further workup. FISH testing for FIP1L1-PDGFRA, PDGFRB, FGFR1, and BCR-ABL1 was negative. Next-generation sequencing (NGS) identified three variants of uncertain significance: CUX1 c.2320G > A (p.Gly774Ser), JAK2 c.2324 C > T (p.Pro775Leu), and CALR c.341 C > G (p.Pro114Arg). None of these variants have been previously reported in association with myeloid neoplasms, and their clinical significance remains unknown. Overall, molecular testing did not support a diagnosis of myeloproliferative neoplasm, favoring a reactive eosinophilia in the setting of systemic inflammatory disease.

#### Pulmonary

The patient’s lung involvement was studied with a high-resolution CT thorax. Results revealed a progression of diffuse interlobular septal thickening alongside ground-glass and peribronchial thickening. A small pericardial effusion with thickening was also appreciated.

#### Cardiac

**Fig. 1:**
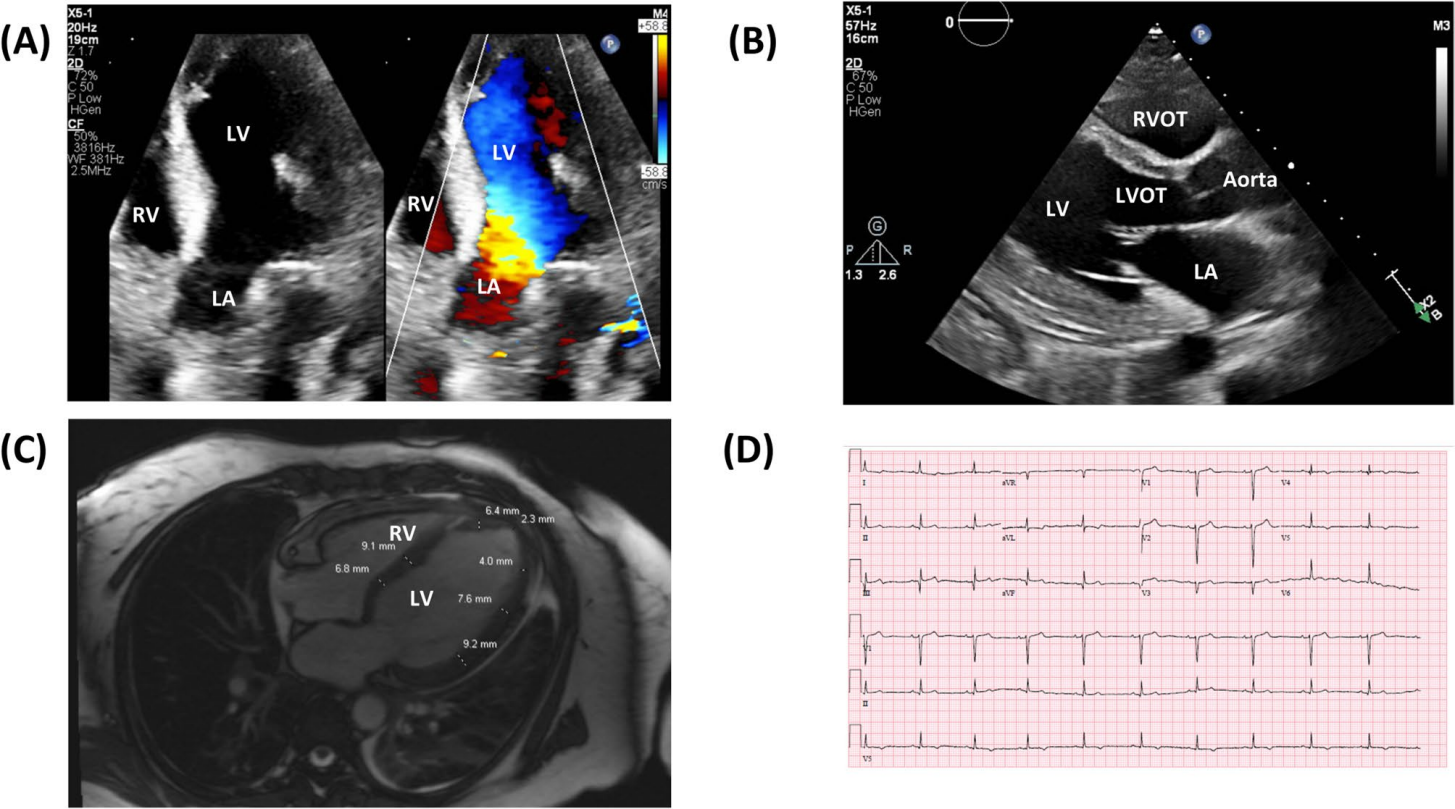
TTE showing mildly dilated left ventricle, with mildly increased wall thickness, eccentric remodeling, and global hypokinesis with moderately reduced systolic function estimated at EF 35–40%, additionally there is an echogenic pendenculated mobile mass (1.5 × 1.6 cm) on the lateral wall of LV (**A**). Again, TTE showing left ventricle mildly dilated with slightly increased wall thickness, eccentric remodeling (**B**). Cardiac MRI showing moderately dilated left ventricle with severe hypokinesis, evidence of infiltrative nonischemic cardiomyopathy (**C**). EKG showing normal sinus rhythm with T-wave abnormalities and potential prior anterior and inferior infarctions (**D**)

Due to the patient’s history of hypertension alongside elevated troponin and NT-pro BNP levels, an EKG and transthoracic echocardiogram (TTE) were ordered. EKG showed normal sinus rhythm with T-wave abnormalities and potential prior anterior and inferior infarctions. TTE revealed a mildly dilated left ventricle with evidence of wall thickening and eccentric remodeling. The left ventricular ejection fraction was reduced at 35–40% with moderate global hypokinesis. Additionally, a pedunculated mobile mass (1.5 × 1.6 cm) was found on the lateral wall of the left ventricle. Cardiac MRI with and without contrast did not show an intracardiac mass, but identified infiltrative nonischemic cardiomyopathy with acute inflammatory edema and hyperemia involving both the left and right ventricular walls. Notably, no areas of delayed gadolinium enhancement, increased extracellular volume, or endocardial fibrosis were observed on contrast-enhanced sequences (Figure 1).

An endomyocardial biopsy was performed to identify the etiology of the cardiomyopathy. Results showed the endomyocardium with mild interstitial mononuclear cell infiltrate and reactive endothelial cells in a background of mildly hypertrophic and vacuolated cardiomyocytes. An aggregate of a few degranulated eosinophils was appreciated, although it should be noted that this low level of eosinophils could have been affected by the patient starting steroid treatment several days prior to undergoing the biopsy.

#### Neurological

Due to the presence of polyneuropathy, nerve conduction velocity (NCV) studies were ordered. The results of this study indicated reduced amplitude from the left peroneal nerve, left tibial motor nerve, bilateral superficial peroneal sensory nerves, and bilateral sural sensory nerves. No response was recorded from the bilateral peroneal with recording from the extensor digitorum brevis (EDB) motor nerve. These findings confirmed the diagnosis of mononeuritis multiplex in the patient.

**Fig. 2:**
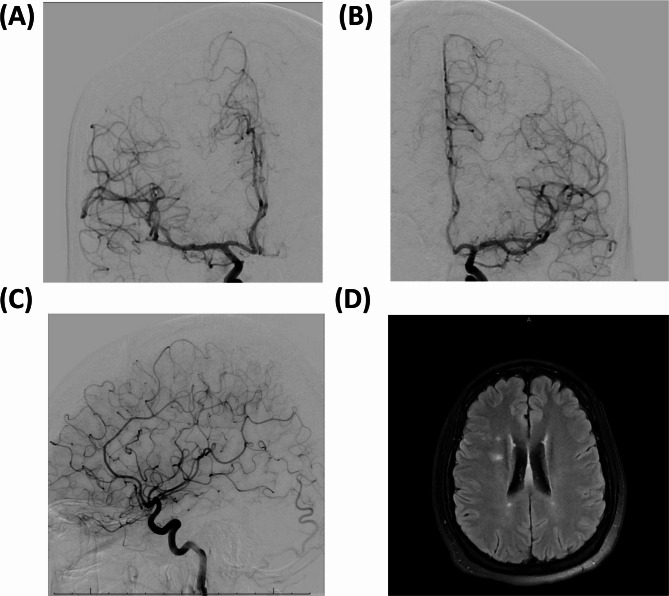
Right ICA AP injection showing focal and multifocal segmental narrowing of both A3 and A4 segments of ACA and M3 and M4 segments of MCA (**A**), Left ICA AP injection also showing decreased caliber of distal branches of both ACA and MCA(**B**), Left ICA Lateral injection again showing decreased caliber of distal branches of both ACA and MCA (**C**), MRI T2 flair showing hyperintensities representing presumed vasculitis related infarcts in bilateral hemispheres (**D**)

A sudden endorsement of vision changes midway through the patient’s hospitalization in the form of floaters and blurriness in the lateral and lower visual fields warranted neurology consultation and stroke workup. The patient was empirically started on aspirin 81 mg daily for secondary stroke prophylaxis and duloxetine 60 mg daily for management of peripheral neuropathy. Cardiac etiologies for embolic stroke were ruled out with 12-lead EKG showed normal sinus rhythm without arrhythmia, and a TTE which revealed no evidence of atrial thrombus, septal defect, or valvular abnormality. Additionally, there was no history of atrial fibrillation, and telemetry monitoring during admission did not reveal arrhythmic episodes. The patient had no personal or family history of hypercoagulability, and lipid panel and HbA1c levels were within normal limits, making atherosclerotic stroke less likely. CT head and computed tomography angiography (CTA) head showed no acute intracranial abnormality. MRI brain without contrast showed numerous areas of hyperintensity with restricted diffusion believed to represent vasculitis-related cortical and white matter track infarcts (Fig. 2). These findings were confirmed on diffusion weighted imaging (DWI).

Due to persistent visual disturbance and lack of improvement, conflicting results between CTA head/neck and MRI studies, and lack of novel high-resolution vessel wall imaging (HR-VWI) MRI imaging capability, digital subtraction angiography (DSA) was performed to rule out microhemorrhage or other acute stroke. DSA was performed using a Siemens Artis Q biplane flat-panel angiography system. Selective catheterization was achieved using a 4-French angled Berenstein catheter and a 0.035-inch Terumo Glidewire. Contrast injections were delivered at rates of 3–6 mL/s depending on vessel size, with injection volumes ranging from 8 to 12 mL per site. DSA revealed decreased caliber of distal branches of both A3 and A4 branches of the right anterior cerebral artery (ACA), M3 and M4 branches of the right middle cerebral artery (MCA).

#### Differential diagnosis

The differential diagnosis at the beginning of the presentation was broad, including eosinophilic pneumonia, eosinophilic leukemia, hypereosinophilic syndrome, medication-induced eosinophilia, EGPA, granulomatosis with polyangiitis (GPA, formerly Wegener’s granulomatosis), or infection due to tuberculosis, aspergillus, or parasites. After review of the patient’s medications, no cause of medication-induced eosinophilia could be identified. Eosinophilic leukemia was ruled out by a negative bone marrow biopsy, and infection was ruled out by negative results across blood cultures and specific parasitic, fungal, and viral testing. Thus, further attention was placed on the possibility of vasculitis. Lack of kidney involvement additionally pointed away from GPA and toward EGPA as the likely etiology. The patient met all six 1990 ACR criteria, which encompassed asthma, eosinophilia, neuropathy, pulmonary infiltrates, and extravascular eosinophils, confirming EGPA. Additionally, the patient scored + 11 on the 2022 ACR criteria, presenting with obstructive airway disease, mononeuritis multiplex, blood eosinophil count ≥ 1 × 109 /L, and biopsy results indicating extravascular eosinophilic-predominant inflammation.

### Treatment

Initially, the patient was treated with intravenous (IV) methylprednisolone 500 mg BID for three days, followed by a gradual oral prednisone taper. Given the severity of her EGPA, six cycles of cyclophosphamide infusion at 15 mg/kg with MESNA were administered for remission induction, with the first three doses 2 weeks apart, and the final 3 doses administered 3 weeks apart [11].

The patient endorsed significant improvement in shortness of breath within days of starting therapy and moderate improvement in mononeuritis multiplex after the first week. Leukocytosis and eosinophilia both normalized shortly after the initiation of steroids without the presence of significant myelosuppression. The patient remained in remission following cyclophosphamide induction and the tapering of steroids, and was placed on azathioprine (AZA) for maintenance therapy. At four-month follow-up, she remained stable with no evidence of disease recurrence or new neurologic or cardiopulmonary symptoms.

## Methods: Systematic review of the literature

### Search strategy

**Fig. 3:**
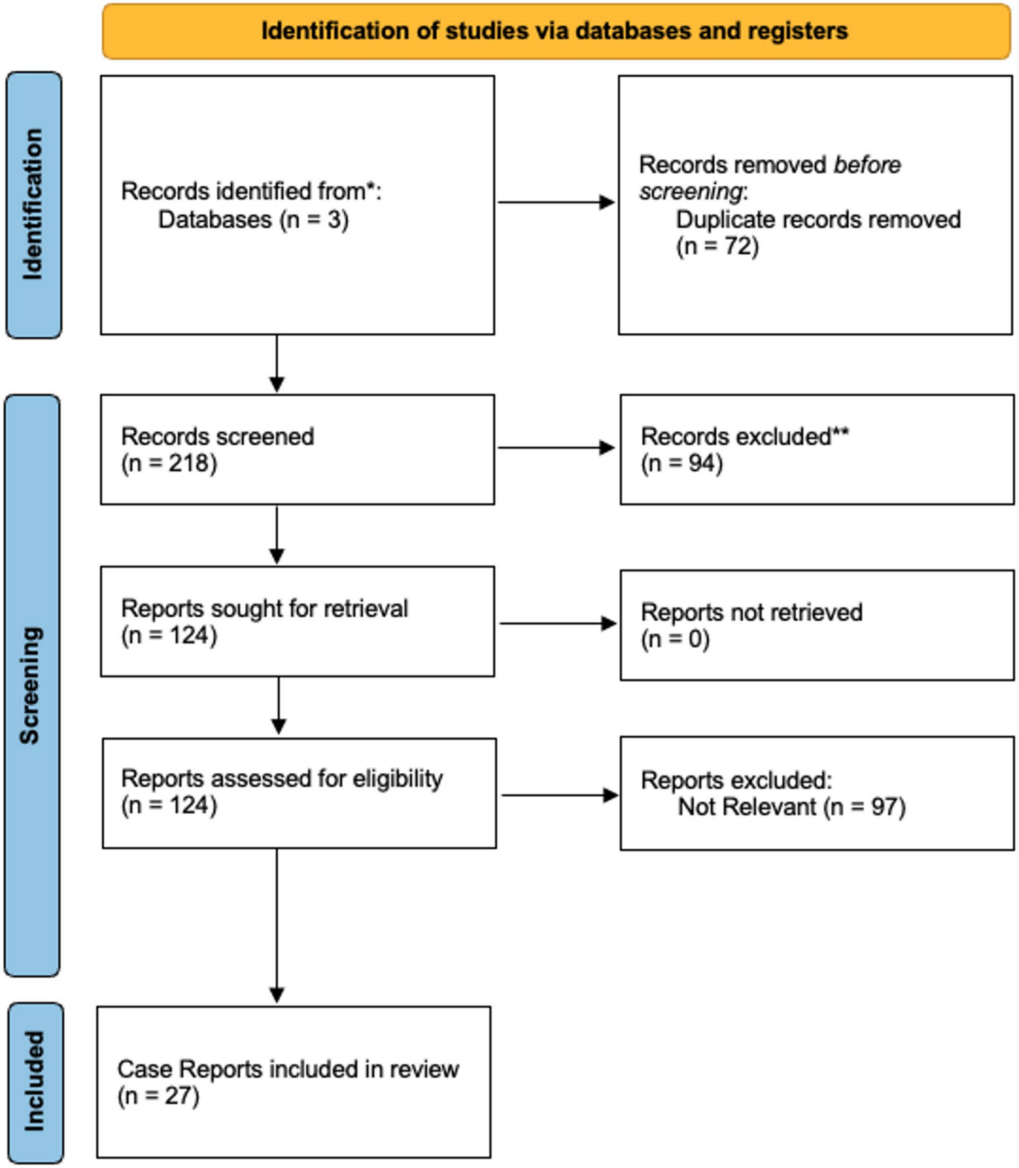
PRISMA flow diagram for systematic literature search of EGPA cases with CNS involvement

A systematic review was conducted using PubMed, Science Direct and Scopus databases up to September 2023 following following the Preferred Reporting Items for Systematic Reviews and Meta-Analyses (PRISMA) guidelines with MeSH search terms: (“Churg-Strauss syndrome” OR “eosinophilic granulomatosis with polyangiitis”) AND (“central nervous system” OR “cerebrovascular”) (Fig. 3). Our search terms and selection criteria are defined below and were not registered in PROSPERO. Case reports and case series of patients with the diagnoses of EGPA with CNS involvement were eligible for inclusion. The exclusion criteria included non-English publications, review articles, correspondences, commentaries, book chapters, studies involving animals, opinion pieces, as well as systematic reviews and meta-analyses with no original clinical case reports.

### Data extraction

Three authors (S.O., V.V., R.G.) independently performed database searches, followed by title, abstract, and finally, full-text reviews to determine inclusion through a pre-determined criterion. Bibliographies of included articles were examined to identify additional articles the original search could have missed. Variables extracted included patient demographics, neurological presenting signs and symptoms, imaging findings, treatment, and outcome.

The screening process was independently conducted by three authors (S.O., V.V., R.G.) following the predefined search strategy and utilizing a data extraction form developed by the research team. Initially, articles were screened based on titles, followed by abstract and full-text review to assess eligibility. Three authors (S.O., V.V., R.G.**)** subsequently evaluated the full texts to finalize article inclusion and extract pertinent data. The bibliographies of included articles were also reviewed for additional relevant studies. Disagreements were resolved through consensus meetings. The selected articles were assessed using the established data extraction methodology, focusing on key variables such as: (1) demographics; (2) neurological presenting signs and symptoms; (3) imaging findings; (4) treatment; and (5) outcome. Individual patient data was extracted from each study in order to be included in the analysis.

### Statistical analysis and summary of literature

Data from the included case reports were organized into a structured table and synthesized descriptively due to the qualitative and heterogeneous nature of the available evidence. Variables such as age, sex, neurological symptoms, imaging modality and findings, treatment type, and outcomes were tabulated to identify trends and differences across cases. Due to the small sample size and variability in reporting, formal meta-analysis or statistical testing was not performed. Instead, we calculated frequencies for categorical variables and means with standard deviations for continuous variables. Imaging modality performance was assessed based on the presence or absence of vascular findings in each case, allowing for comparative insights into diagnostic utility across DSA, MRA, CTA, and other techniques.

## Results: Systematic review of the literature

### Electronic search yield

Our literature review yielded 290 citations. Upon initial screening, we excluded 94 citations for irrelevance, and another 97 were excluded after full article evaluation. Additionally, we identified 72 duplicate records. Ultimately, we included 27 case reports in our review from October 2001 to August 2023, each detailing a unique patient diagnosed with EGPA complicated by CNS involvement [12–38] (Fig. 3; Table 2).

**Table 2:** Results from systemic literature review of EGPA with CNS involvement, with the reference, age in years, sex, neurological symptoms, imaging, imaging findings, management, and outcomes discussed in the 27 included case reports

Reference	Date	Age (y)/Sex	Neurological Symptoms	Imaging: Finding	Management	Outcome
Klearchos Psychogios et al. [12]	March 2017	63/F	Psychomotor retardation, right facial and right arm paresis	MRI: Bihemispheric infarctions	Anticoagulation	Unspecified
				MRA: None		
Yumika Saito et al. [13]	December 2018	59/M	Left foot paraesthesia, altered consciousness	MRI: Bihemispheric infarctions	Glucocorticoid, Cyclophosphamide, and Cilostazol	Clinical Improvement
				MRA: None		
Meng-Ju Cheng et al. [14]	December 2021	60/M	Right hemiparesis	MRI: Bihemispheric infarctions	Methylprednisolone, Prednisolone, Cyclophosphamide, Anticoagulation	Clinical Improvement
Koji Tanaka et al. [15]	January 2012	77/F	Truncal ataxia	MRI: Right hemisphere of the cerebellum infarction	Corticosteroids	Clinical Improvement
Jasemine Saech et al. [16]	September 2009	46/M	Right hemiparesis	MRI: Thalamus and pons infarctions	Cyclophosphamide, Steroids, Acetylsalicylic Acid, Rituximab	Recurrance
Mirjana Arsenijević et al. [17]	June 2021	57/F	Paresthesia, dysesthesia, and allodynia in extremities	CT: Bilateral basal ganglia infarctions	Methylprednisolone, Anticoagulation,, Prednisone	Recurrance
Pooia Fattahi et al. [18]	January 2015	53/M	Peripheral neuropathy, dysarthria, left facial weakness, left upper extremity sensory loss, left hemiparesis	MRI: Right anterior centrum semiovale infarctions, right basal ganglia hemorrhage	Anticoagulation, Hydrocortisone	Clinical Improvement
				CT: Right caudate nucleus infarction		
				CTA: None		
Ken-Ei Sada et al. [19]	September 2021	62/F	Lower extremity peripheral sensory neuropathy	MRI: Occipital lobe infarctions	Prednisolone, Mepolizumab	Clinical Improvement
Tessa A Harland et al. [20]	April 2019	48/F	Altered mental status, dysarthria, lower extremity weakness, paresthesia	CT: Posterior parieto-occipital hemorrhage	Rituximab, Steroids	Death
				MRI: Posterior parieto-occipital hemorrhage		
				A: Bilateral anterior cerebral artery stenosis		
Hiroki Ezaki et al. [21]	January 2021	29/M	Altered mental status	CT: Hematoma extending to the ventricle from the left putamen	Cyclophosphamide, Prednisolone	Clinical Improvement
				CTA: None		
Alison Lee Huckenpahler et al. [22]	April 2022	69/M	Right arm weakness and numbness	MRI: Periventricular infarctions	Methylprednisolone, Prednisone, Rituximab	Clinical Improvement
				CTA: None		
Yuki Ueta et al. [23]	December 2020	75/M	Right sided hemiplegia, right sided extremity weakness	MRI: Left posterior limb of the internal capsule infarction	Corticosteroids	Death
Dingkang Xu et al. [24]	March 2019	38/M	Intermittent right frontal headache, impaired extension of the right upper extremity	MRI: Temporal and occipital lobe demylinating lesions	Methylprednisolone, Prednisone, Cyclophosphamide	Death
Samwel Sylvester Msigwa et al. [25]	December 2020	81/F	Left leg weakness, lower extremity paresthesia	CT: Bilateral paraventricular and bilateral basal ganglia infarctions	Corticosteroids	Clinical Improvement
Anusha Challa et al. [26]	July 2023	61/F	Flaccid quadriplegia, bulbar weakness, bilateral facial palsy, diffuse areflexia distal limb loss of vibration and proprioception	MRI: Left internal capsule infarction	Prednisolone, Cyclophosphamide	Clinical Improvement
				A: None		
Vinoth Doraiswamy et al. [27]	March 2022	14/F	Headache	MRI: Subacute infarctions	Methylprednisolone, Rituximab, Lisinopril, Metoprolol, Diuretics, and Acitrom	Clinical Improvement
				MRA: None		
				MRV: None		
Gustav Mattsson et al. [28]	December 2017	53/M	Dysphagia, dysarthria	CT: Left parietal lobe hemorrhage, right frontal lobe infarction	Methylprednisolone, Prednisolone, Cyclophosphamide	Clinical Improvement
				A: Vertebral artery stenosis		
Lucrezia Mencarelli et al. [29]	August 2023	46/F	Left leg dystonia, Right extrapyramidal syndrome	MRI: Bihemispheric infarctions	Methylprednisolone, Glucocorticoids, Intravenous Immunoglobulin, Romiplostim, Benralizumab	Clinical Improvement
Vitalie Văcăraș et al. [30]	January 2021	79/F	Altered mental status, extremity weakness, babinski sign on the right, left arm dysmetria with hypermetria, right hemihypoesthesia, lower limb paresthesia	CT: Left sylvian artery and bilateral periventricular locations infarctions	Prednisone, Azathioprine, Cerebrolysin, Sodium Chloride, Anticoagulants, Proton Pump Inhibitors, Amlodipine, Carvedilol	Clinical Improvement
				MRI: Periventricular semioval center and bilateral subcortical infarctions		
Toshikazu Mino et al. [31]	September 2021	69/F	Barré sign of the left upper extremity with pronation, left extremity weakness	MRI: Bihemispheric infarctions	Prednisolone	Clinical Improvement
				MRA: None		
Halil Yildiz et al. [32]	February 2016	75/M	Dysarthria, extremity weakness, presyncope	MRI: Bihemispheric infarction and hemorrhage	Methylprednisolone, Cyclophosphamide	Clinical Improvement
				CT: Bihemispheric infarction and hemorrhage		
				A: None		
Marino Paroli et al. [33]	November 2012	46/F	Extremitie sensorimotor deficis	MRI: Left thalamic infarction	Corticosteroids, Prednisone, Cyclophosphamide, Anticoagulants	Clinical Improvement
Niccolò E. Mencacci et al. [34]	February 2011	29/M	Global aphasia, right emiparesis, omolateral Babinski at plantar cutaneous stimulation, right sided atrophy, reduction of supinator and achilles tendon reflex at right limbs	CT: Left straitum nucleus haemorrhage iinvolving the temporal lobe, with intraventricular extension	Methylprednisolone, Prednisone	Clinical Improvement
				A: None		
Maria Teresa Sartori et al. [35]	July 2006	40/F	Extremity paresthesia, decreased visual acuity in the right eye	MRI: Bilateral parietal haemorrhages	Betamethasone, Prednisone, Anticoagulants	Clinical Improvement
				CT: Bilateral parietal haemorrhages		
				MRV: Superior sagittal sinus and right lateral sinus thrombosis		
Dong-Wha Kang et al. [36]	October 2001	22/M	Aphasia, right hemiparesis	MRI: Left MCA territory infarction	Prednisone	Clinical Improvement
				A: Left MCA branch stenosis		
Shigeyuki Sakamoto et al. [37]	September 2004	36/F	Severe headache and vomiting	CT: Vertebral artery subarachnoid hemorrhage	Prednisone	Clinical Improvement
				A: Right intracranial vertebral artery dissecting aneurysm, basilar artery stenosis		
Leyli Ghaeni et al. [38]	January 2010	77/F	Hypotonic tetraparesis, global aphasia	MRI: Bihemispheric infarctions	Cyclophosphamide, Methylprednisolone	Clinical Improvement
				CTA: None		

F = Female, M = Male, MRI = magnetic resonance imaging, MRA = magnetic resonance angiography, MRV = Magnetic Resonance Venograph, CT = computed tomography, A = Angiogram

### Systematic review results

Across the 27 patients included, mean patient age was 54.22 years with a standard deviation of 18, split between 14 females and 13 males. The most common neurological symptoms were extremity weakness (*n* = 8), paresthesia (*n* = 5), hemiparesis (*n* = 4), dysarthria (*n* = 4), and altered mental status (*n* = 3).

Various imaging modalities for identifying CNS pathology were used, including MRI (*n* = 21), CT (*n* = 11), angiogram (*n* = 8), MRA (*n* = 4), CTA (*n* = 4), and MRV (*n* = 2). Angiograms specifically were used to identify stenosis of bilateral ACA in *Tessa A Harland et al.*, the vertebral artery (VA) in *Gustav Mattsson et al.*, an MCA branch in *Dong-Wha Kang et al.*, and the basilar artery (BA) in *Shigeyuki Sakamoto et al.* [20, 28, 36, 37] Additionally, *Shigeyuki Sakamoto et al.* also visualized a right intracranial VA dissecting aneurysm using the same modality [37]. Three of the included studies had no findings using angiography in EGPA patients presenting with CNS involvement [26, 32, 34]. In the four cases that used magnetic resonance angiography (MRA) for imaging, no vascular anomalies were visualized with that modality [12, 13, 27, 31]. Likewise, in the four cases that used CTA for imaging, no vascular anomalies were visualized with that modality [18, 21, 22, 38]. MRV was used by both *Vinoth Doraiswamy et al.*, who identified no vascular anomalies with that modality, and *Maria Teresa Sartori et al.* who visualized thrombosis of the superior sagittal sinus and right lateral sinus [27, 35]. 

Treatment most commonly involved methylprednisolone (*n* = 14), followed by cyclophosphamide (*n* = 11), anticoagulation (*n* = 10), and prednisolone (*n* = 10). Two cases described recurrence of EGPA symptoms following treatment: *Jasemine Saech et al.* with thalamic and pontine infarcts identified on MRI, and *Mirjana Arsenijević et al.* with bilateral basal ganglia infarcts identified on CT [16, 17]. Three cases of death were reported: *Yuki Ueta et al.* with left posterior limb of the internal capsule infarct identified on MRI, *Dingkang Xu et al.* with temporal and occipital lobe demyelinating lesions identified on MRI, and *Tessa A Harland et al.* with posterior parieto-occipital hemorrhage identified on CT and MRI, along with the previously mentioned bilateral ACA stenosis identified on angiogram [20, 23, 24].

## Discussion

### Epidemiology and management

EGPA has an estimated annual incidence between 0.11 and 2.6 new cases per million, with a prevalence of 10.7–14 per million adults [39]. In our presentation, we show a severe case with both CNS involvement and cardiomyopathy.

EGPA, when left untreated, results in a 50% mortality rate at three months [40, 41]. As such, the induction of the correct treatment regimen is imperative. The standard treatment for patients with active non-severe disease is glucocorticoids along with either mepolizumab, methotrexate (MTX), AZA, mycophenolate mofetil (MMF), or rituximab (RTX) [11]. However, for patients presenting with active severe disease, the combination of IV pulse or high glucocorticoids along with either cyclophosphamide or RTX, is recommended [11]. In this instance, the patient exhibited both cardiomyopathy and CNS involvement indicating active severe disease. Therefore, we initiated a three-day course of pulsed methylprednisolone, followed by a prednisone taper and multiple cycles of cyclophosphamide, based on the presence of cardiac involvement, five-factor score, and negative ANCA titers.

Classically, histological examination in EGPA reveals eosinophilia, necrotizing vasculitis, and eosinophil-rich necrotizing granulomas [42]. Cardiac biopsy is a valuable tool for diagnosing eosinophilic cardiomyopathy in patients with EGPA. In our patient, initial cardiac dysfunction was detected via TTE, which revealed a reduced ejection fraction, concentric remodeling, and a mobile left ventricular mass. Subsequent cardiac MRI identified a nonischemic infiltrative cardiomyopathy characterized by myocardial edema and hyperemia; however, it did not demonstrate late gadolinium enhancement (LGE), increased extracellular volume (ECV), or endocardial fibrosis typically seen in eosinophilic cardiomyopathies such as Löffler endocarditis [43–45]. These atypical MRI findings, particularly in the absence of ANCA positivity, posed a diagnostic challenge. Histopathological confirmation was ultimately provided by endomyocardial biopsy, which showed mildly hypertrophic and vacuolated cardiomyocytes with interstitial mononuclear infiltrates and degranulated eosinophils within the endocardium, despite the confounding effect of prior steroid induction. This case illustrates the complementary roles of imaging and tissue diagnosis in EGPA. While cardiac MRI played a pivotal role by detecting inflammation that might have been missed on conventional imaging, biopsy served as a critical confirmatory tool in the absence of classical MRI features. Prior literature underscores the superiority of cardiac MRI over traditional imaging for tissue characterization and inflammation detection, particularly in ANCA-negative phenotypes [46–48]. Therefore, integrating cardiac MRI early in the diagnostic workup, followed by biopsy in inconclusive cases, may improve detection of myocardial involvement in EGPA and guide appropriate immunosuppressive therapy.

ANCA has been found to be positive in about 40% of cases, more frequently observed in the small vessel vasculitis phenotype [49]. The symptoms commonly seen in these individuals include mononeuritis multiplex, muscle pain, shifting joint pain, and the appearance of crescentic or necrotizing glomerulonephritis [50]. ANCA-negative patients tend to have an eosinophilic phenotype, as well as a higher incidence of myocarditis [49]. Our report of an ANCA-negative patient thus lends to this trend of myocardial involvement. It is worth noting, however, that mononeuritis multiplex and vasculitis are more commonly associated with ANCA positivity.

Emerging evidence suggests that eosinophilia, particularly in ANCA-negative EGPA, may independently contribute to stroke pathogenesis through multiple prothrombotic and cytotoxic mechanisms. Eosinophils can activate platelets and upregulate tissue factor via granule proteins such as eosinophil cationic protein (ECP) and major basic protein (MBP), creating a hypercoagulable state [51]. These proteins, along with eosinophil-derived ROS and extracellular traps, damage the endothelium and promote thrombus formation [51, 52]. In ANCA-negative patients, eosinophilic myocarditis and endocardial fibrosis can facilitate intracardiac thrombus formation, predisposing to cerebral embolism, even in the absence of frank vasculitis [53]. Autopsy findings from *Hira et al.* corroborate this dual mechanism: the patient was found to have multiple cerebral infarcts from both cardiogenic embolism due to mural thrombi and direct eosinophilic vasculitis of cortical vessels, despite lacking classic EGPA features like asthma [54]. In our patient, the combination of ANCA-negativity, marked eosinophilia, cardiomyopathy, and multifocal infarcts supports eosinophil-mediated thromboembolism as a likely contributor. These mechanisms underscore the need to consider early cardiac imaging and anticoagulation in similar cases.

In individuals experiencing mononeuritis multiplex, the peroneal nerve is often affected first, compared to the less common tibial, ulnar, median, and radial nerves [55]. In our case, the sensory nerve conduction study showed low amplitude in the left and right peroneal nerves, left and right superficial peroneal nerves, left tibial nerves, and the left and right sural nerves.

### Cerebrovascular imaxging

Previous literature has reported angiographic imaging as ineffective for identifying CNS involvement for EGPA [13]. Indeed, in three of the studies included in our systematic review, no positive findings were found on angiography [26, 32, 34]. Additionally, most authors that describe CNS involvement with EGPA, including cerebral infarcts, rarely give a description of the involved vessels [38]. However, we propose that there is utility in angiography, as seen in the majority (4 out of 7) of the cases found in our systematic review involving angiographic imaging [20, 28, 36, 37]. The type of angiography matters: CTA and MRA had no positive findings in any case, giving credence to interventional radiology (IR) angiography, such as digital subtraction angiography, as a more effective imaging modality for cerebrovascular involvement of EGPA. In the four cases with positive cerebrovascular findings, the bilateral ACA, VA, MCA branch, and BA were reported as having stenosis resulting from EGPA vasculitis. In our case, we demonstrated involvement of the right MCA and ACA, which was not initially identified on CTA, indicating a lower sensitivity of CTA that was compensated for by angiographic imaging. The confirmatory DSA allowed for the categorization of severe EGPA by the FFS, ultimately changing the patient’s treatment plan and prognosis. This finding correlates with literature reporting negative results on CTA, even in the presence of stenosis on angiography [18, 21, 22, 38]. Thus, we propose that DSA be used more liberally as a sensitive and specific diagnostic tool in patients with cerebrovascular complications of vasculitis when conventional imaging methods are inconclusive.

While DSA offers unparalleled spatial resolution for detecting luminal stenosis, it does not permit direct assessment of vessel wall pathology and its sensitivity in diagnosing CNS vasculitis varies widely. Several other conditions can produce intracranial arterial stenoses that mimic CNS vasculitis on angiography. Atherosclerosis is the most common cause, often affecting large proximal vessels with eccentric, calcified plaques [56]. Other systemic vasculitides, such as primary angiitis of the central nervous system (PACNS), GPA, and polyarteritis nodosa (PAN), can present with multifocal stenoses similar to EGPA [57, 58]. Infectious etiologies including varicella-zoster virus (VZV), syphilis, tuberculosis, and fungal meningitis may also cause inflammatory vasculopathy and stenosis [57]. Non-inflammatory mimics such as reversible cerebral vasoconstriction syndrome (RCVS) and Moyamoya disease should also be considered, as they can present with multifocal or progressive arterial narrowing [57, 59]. Therefore, cerebrovascular imaging must be interpreted in the context of systemic symptoms, laboratory findings, and biopsy results when available, to avoid misclassification and guide appropriate management.

Prior literature reports DSA sensitivity ranging from 27 to 90% and specificity as low as 30%, largely due to its inability to distinguish between various causes of luminal irregularities, such as reversible cerebral vasoconstriction syndrome, intracranial atherosclerosis, and true vasculitis [60]. MRI-based HR-VWI has thus emerged as a powerful non-invasive tool, enabling direct visualization of mural inflammation, such as concentric wall thickening and enhancement, which improves sensitivity and specificity for vasculitis [61]. HR-VWI abnormalities have been shown to correlate with multifocal angiographic stenoses and yield a sensitivity of 94% and specificity of 95% for diagnosing CNS vasculitis. This technique can also help differentiate autoimmune from infectious vasculopathies, including neurosyphilis and varicella-zoster virus, which often mimic vasculitis in both clinical and angiographic presentations [52]. Although CTA and MRA are frequently employed in initial evaluations due to their accessibility, prior studies demonstrate their limited sensitivity in small- and medium-vessel vasculitis; they often fail to detect subtle mural or distal branch abnormalities [62]. As such, while DSA remains a valuable modality, especially when HR-VWI is unavailable, our findings underscore the complementary value of HR-VWI in the diagnostic algorithm for CNS vasculitis, particularly when non-invasive angiographic studies are inconclusive or discordant with clinical suspicion [61].

Beyond the CNS, vascular imaging plays a critical role in identifying extracranial vasculitic complications in EGPA. Coronary angiography, for instance, has revealed rare but life-threatening manifestations such as spontaneous coronary artery dissection (SCAD) and refractory coronary vasospasm, as reported in a patient who experienced recurrent acute coronary syndromes despite normal initial angiograms [63]. These vascular complications were attributed to eosinophilic inflammation, confirmed after elevated eosinophil counts and EGPA diagnosis. Additionally, cross-sectional vascular imaging modalities such as CTA and MRA have successfully identified mural thickening and stenosis in systemic arteries, including mesenteric, renal, and iliac vessels, indicative of medium-vessel vasculitis in multisystem EGPA involvement [62]. While not definitive in isolation, such findings become diagnostically valuable when integrated with systemic symptoms suggestive of vasculitis. In our patient, the lack of vascular abnormalities on CTA prompted escalation to DSA, which ultimately uncovered stenoses of the ACA and MCA branches. This parallels extracranial patterns seen in systemic EGPA, underscoring the adjunctive diagnostic utility of angiographic imaging beyond the CNS, particularly when conventional modalities yield inconclusive results in the setting of suspected multi-organ involvement.

While DSA is traditionally used to evaluate arterial pathology in CNS vasculitis, its ability to assess venous sinuses expands its diagnostic utility in EGPA, particularly for detecting cerebral venous sinus thrombosis (CVST). Although rare, CVST has been increasingly recognized as a life-threatening manifestation of EGPA. For example, *Tanomogi et al.* described a fatal case of CVST in a 44-year-old woman with EGPA, in which thrombosis extended from the internal jugular vein to the superior sagittal sinus and was only diagnosed after clinical deterioration [64]. Similarly, *Ananth et al.* reported transverse sinus thrombosis in a 17-year-old male with EGPA, diagnosed via CT brain imaging [65]. These cases underscore the need for vigilance in patients presenting with neurologic symptoms and eosinophilia. Importantly, DSA, magnetic resonance venography (MRV), and other angiographic tools should be considered when symptoms such as headache, altered mental status, or thrombocytopenia arise, even if initial CT or MRI findings are non-revealing. Thus, angiographic imaging provides crucial insights into both arterial and venous cerebrovascular pathology in EGPA, guiding timely intervention and potentially improving outcomes.

### Limitations

This study is limited by the inherent constraints of a single case report and the rarity of EGPA with CNS involvement, which may limit the generalizability of our findings. While DSA provided key diagnostic insights in this case, it was not directly compared to HR-VWI due to availability at our institution, which may offer complementary or superior information in differentiating vasculitic from non-vasculitic etiologies. Additionally, although our systematic literature review followed PRISMA guidelines and included multiple databases, the inclusion of only English-language case reports may introduce selection bias and overlook relevant non-English publications. Furthermore, variability in imaging modalities, diagnostic criteria, and treatment regimens across cases complicates the ability to draw definitive conclusions about optimal diagnostic or management strategies. Finally, as a retrospective analysis, we cannot assess the longitudinal utility of DSA or its prognostic implications in EGPA with cerebrovascular involvement.

## Conclusion

Angiographic imaging has a significant role in diagnosing cerebrovascular involvement in EGPA, a rare complication often elusive through other modalities. While IR angiography demonstrated positive findings in most cases within our systematic review, CTA and MRA yielded few positive results. This underscores the significance of IR angiography in uncovering cerebrovascular implications, such as stenosis affecting vessels including the ACA, VA, MCA, and BA. Our presentation further contributes by revealing previously unreported involvement of the right MCA and ACA through angiography, reaffirming the limited sensitivity of CTA in detecting these cerebrovascular manifestations. In the context of EGPA, our findings emphasize a potentially novel role of angiographic imaging for a comprehensive assessment of cerebrovascular involvement, offering valuable insights into this rare but impactful aspect of EGPA.

## Data Availability

Data supporting the findings of this case report and literature review are available within the article or by request to the corresponding author.

## Abbreviations

EGPA: Eosinophilic granulomatosis with polyangiitis
CSS: Churg-Strauss syndrome
CNS: Central nervous system
CT: Computed tomography
MRI: Magnetic resonance imaging
DSA: Digital subtraction angiography
ACA: Anterior cerebral artery
MCA: Middle cerebral artery
MRA: Magnetic resonance angiography
CTA: Computed tomography angiography
MRV: Magnetic resonance venography
ANA: Antinuclear antibody
CRP: C-reactive protein
UTMB: University of Texas Medical Branch
TTE: Transthoracic echocardiogram
EKG: Electrocardiogram
NCV: Nerve conduction velocity
MPO: Myeloperoxidase
ANCA: Antineutrophil cytoplasmic antibodies
AZA: Azathioprine
MTX: Methotrexate
MMF: Mycophenolate mofetil
HR-VWI: High-resolution vessel wall imaging
RTX: Rituximab
FFS: Five Factor Score
IR: Interventional radiology
PACNS: Primary angiitis of the central nervous system
PAN: Polyarteritis nodosa
VZV: Varicella-zoster virus
SCAD: Spontaneous coronary artery dissection
